# A tailored *Cln3*^*Q352X*^ mouse model for testing therapeutic interventions in CLN3 Batten disease

**DOI:** 10.1038/s41598-020-67478-5

**Published:** 2020-06-29

**Authors:** Logan Langin, Tyler B. Johnson, Attila D. Kovács, David A. Pearce, Jill M. Weimer

**Affiliations:** 1 Pediatrics and Rare Diseases Group, Sanford Research, 2301 E. 60th N, Sioux Falls, SD 57104 USA; 2 Department of Pediatrics, Sanford School of Medicine, University of South Dakota, Sioux Falls, SD USA

**Keywords:** Lipid-storage diseases, Experimental models of disease

## Abstract

CLN3 Batten disease (CLN3 disease) is a pediatric lysosomal storage disorder that presents with progressive blindness, motor and cognitive decline, seizures, and premature death. CLN3 disease results from mutations in *CLN3* with the most prevalent mutation, a 966 bp deletion spanning exons 7–8, affecting ~ 75% of patients. Mouse models with complete *Cln3* deletion or *Cln3*^*Δex7/8*^ mutation have been invaluable for learning about both the basic biology of CLN3 and the underlying pathological changes associated with CLN3 disease. These models, however, vary in their disease presentation and are limited in their utility for studying the role of nonsense mediated decay, and as a consequence, in testing nonsense suppression therapies and read-through compounds. In order to develop a model containing a disease-causing nonsense point mutation, here we describe a first-of-its-kind *Cln3*^*Q352X*^ mouse model containing a c.1054C > T (p.Gln352Ter) point mutation. Similar to previously characterized *Cln3* mutant mouse lines, this novel model shows pathological deficits throughout the CNS including accumulation of lysosomal storage material and glial activation, and has limited perturbation in behavioral measures. Thus, at the molecular and cellular level, this mouse line provides a valuable tool for testing nonsense suppression therapies or read through compounds in CLN3 disease in the future.

## Introduction

CLN3 Batten disease (CLN3 disease), also referred to as juvenile neuronal ceroid lipofuscinosis (JNCL), is an autosomal recessive disorder caused by mutations in *CLN3*. It is the most common form of Batten disease with over 40 known pathogenic mutations in *CLN3*^1,2^. Patients typically present with progressive vision loss starting around 5 years of age as well as advanced cognitive and motor decline, development of behavioral problems, seizures, and ultimately death^3^. The most prevalent disease causing *CLN3* mutation, accounting for ~ 75% of all homozygous mutations and ~ 20% of compound heterozygous mutations, is a 966 bp deletion that deletes exons 7 and 8^4^. Additionally, a broad spectrum of splice variants, missense, nonsense and frameshift mutations exist that result in non-syndromic retinal disease, protracted disease course, or the common CLN3 disease progression (reviewed in^5^). Rather than looking at a “one-size-fits-all” approach to therapies, researchers and clinicians are analyzing individual patient genetics to determine whether biologicals, read-through compounds or targeted genetic approaches, like gene therapy^6^ or antisense oligonucleotides^7,8^, can impact disease progression (See reviews^9,3^). Therefore in order to have translational success, it is paramount to have robust animal models in place to develop and test these prospective, tailored therapies of CLN3 disease.

Nonsense mediated decay (NMD) is an evolutionarily conserved quality control pathway in eukaryotic organisms that is responsible for inspecting mRNA for errors and eliminating any error-containing transcripts from the transcriptome, as well as controlling the amount of non-mutated transcript in the transcriptome^10^. NMD typically occurs during the first translation of mutant transcript, where mutant mRNAs that contain premature termination codons (PTC) prohibit the ribosome from reaching and removing the exon junction complex, ultimately triggering the NMD signaling cascade^10^. PTCs can be caused by various mutations, though nonsense point mutations are specifically characterized as creating a PTC through the mutation of a single base pair. Nonsense point mutations are frequent causes of human inherited diseases, accounting for approximately 20% of all disease-causing single base pair mutations^11,12^. While NMD is important for quality control, in the context of human disease NMD may exacerbate disease progression, as some truncated proteins’ partial function may be enough to mitigate or reduce the severity of the disease^13^. As such, NMD is a growing target for therapeutic intervention through the use of read-through compounds, which promote translation through the PTC, or nonsense suppression therapies, which block the NMD signaling cascade, in several disease indications, including Batten disease^14,15^.

Mouse models with complete *Cln3* deficiency or carrying the common 966 bp deletion of exons 7 and 8 have been generated in order to better study and understand CLN3 disease^16,17,20^. While the *Δ7/8* genotype results in a frameshift mutation, 28 novel amino acids, and a PTC^18–20^, the model is not suitable for exploring read-through compounds or nonsense suppression therapies, as the addition of 28 novel amino acids may generate a translated protein with altered function relative to wildtype *Cln3*. However, previous research in a nonsense point mutant CLN3 patient line (c.1054C > T (p.Gln352Ter)) revealed that *CLN3*^*Q352X*^ mRNA expression could be increased via NMD inhibition by siRNA-mediated knockdown, opening the door to study the effects of nonsense suppression and read-through therapies using a relevant CLN3 point mutation.

Here, to expand on testing this therapeutic approach for CLN3 disease, we generated a novel, nonsense point mutant mouse model of CLN3 disease based on the CLN3 patient mutation described above. We report the molecular, behavioral and neuropathological characterization of homozygous *Cln3*^*Q352X*^ mice, showcasing their utility as a model of CLN3 disease and potential for screening nonsense suppression and read-through therapies in the future.

## Results

### Generation of ***Cln3***^***Q352X***^ mice

**Figure 1:**
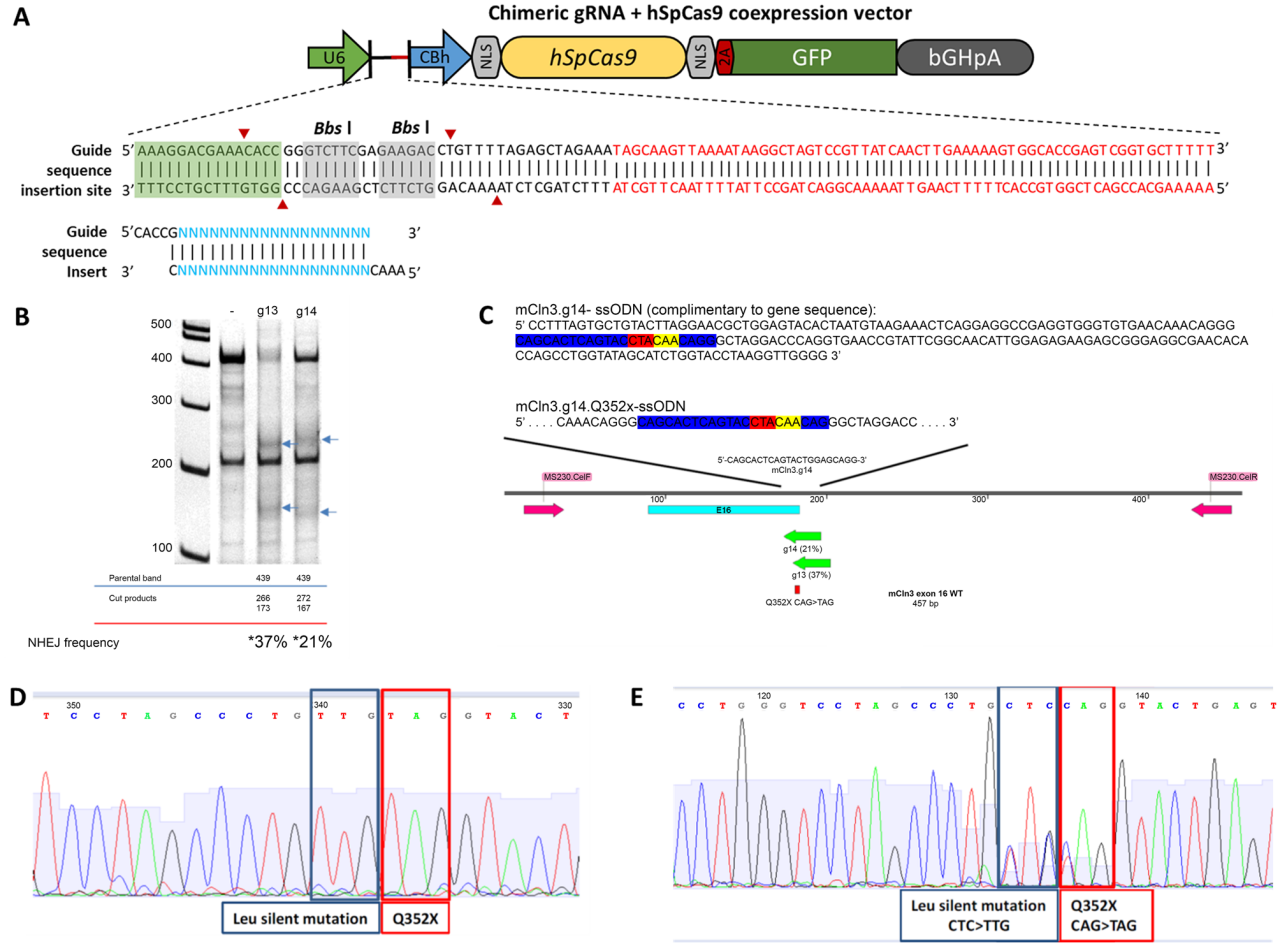
Generation of *Cln3*^*Q352X*^ mice. (**A**) Schematic of targeting vector. Guide RNAs were cloned into a guide RNA/cas9 expression vector by inserting double-stranded oligo cassettes into the *Bbs* I sites. gRNA and cassette sequences are demonstrated in Table 1. Each oligo cassette contained 20 bp gRNA sequences with a guanosine at the 5′ end for optimal expression, and adherent ends for cloning at *Bbs* I sites. (**B**) PCR amplification of transfected mouse N2A cells with each of the two gRNA vectors, and results of SURVEYOR assay showing non-homologous end joining frequency. (**C**) Schematic of single stranded oligo donor nucleotide (ssODN) DNA donor generation using mCln3.g14. Blue: mCln3.g14, Red: Q352X mutation, Yellow: silent leucine mutation. (**D**) Sequence chromatogram of cloned homozygous *Cln3*^*Q352X*^ mouse. (**E**) Representative sequence chromatogram of heterozygous F1 *Cln3*^*Q352X*^ mouse.

*Cln3*^*Q352X*^ mice were generated by Applied StemCell Inc. using CRISPR technology to introduce a nonsense mutation in exon 16 (CAG > TAG), causing glutamine352 (Q) to be replaced with a premature stop codon (X). Two *Cln3* guide RNAs were selected (Table 1), cloned into guide RNA/cas9 expression vectors, and transfected into mouse N2A cells for evaluation of non-homologous end joining efficiency (Fig. 1A,B). While mCLN3.g13 had greater efficiency (37% vs. 21%), mCLN3.g14 was chosen to generate donor DNA due to its optimal position to the point mutation.

**Table 1:** Sequences of guide RNA candidates for *Cln3* modification and double-stranded oligo cassettes.

	Name	Sequence
mCln3.g13	mCln13.g13	5′ACAGGGCAGCACTCAGTACC3′
Oligo cassette	mCln13.g13-pX458-F mCln13.g13-pX458-R	5′CACCGACAGGGCAGCACTCAGTACC3′ 3′CTGTCCCGTCGTGAGTCATGGCAAA5′
mCln3.g14	mCln14.g14	5′CAGCACTCAGTACCTGGAGC3′
Oligo cassette	mCln14.g14-pX458-F mCln14.g14-pX458-R	5′CACCGCAGCACTCAGTACCTGGAGC3′ 3′CGTCGTGAGTCATGGACCTCGCAAA5′

A single stranded oligodeoxylnucleotide donor was made based on mCLN3.g14, with a silent mutation added to leucine351 (CTC > TTG) to prevent the repaired genome sequence from being re-targeted, and combined with mCLN3.g14 Cas-9 vectors and active guide RNA (Fig. 1C). The mCln3-Q352X CRISPR cocktail was injected into the cytoplasm of C57BL/6 embryos, and newborn mice screened for integration. Of the 100 embryos injected, 76 developed and were implanted into three surrogate mice, with 12 mice ultimately born. One cloned male was heterozygous for the *Cln3*^*Q352X*^ mutation and one male was homozygous, as confirmed by sequencing chromatograms (Fig. 1D, homozygous animal). These two animals were used as founders and mated to wildtype females to produce the F1 generation, which produced nine male and four female heterozygous *Cln3*^*Q352X*^ animals that were sequenced, shipped, and bred to generate homozygous *Cln3*^*Q352X*^ mice (Fig. 1E, heterozygous founder).

### *Cln3*^*Q352X*^ mice show reduced *Cln3* transcript levels and variable TPP1 and PPT1 enzyme activities

mRNA was measured in various tissues from wildtype and *Cln3*^*Q352X*^ mice to determine *Cln3* transcript levels. Significantly different *Cln3*^*Q352X*^ transcript levels ranged from 34 to 67% of wildtype and were seen in several tissues of both male and female *Cln3*^*Q352X*^ mice, including the cerebral cortex, thalamus, brainstem, lung, and kidney (Table 2). *Cln3*^*Q352X*^ males also had lower transcript levels in the spleen and liver, whereas *Cln3*^*Q352X*^ female mice displayed decreased transcript levels in the heart and eye.

**Table 2:** *Cln3*^*Q352X*^ mice show lowered *Cln3* transcript abundance and variable PPT1 and TPP1 activity in multiple tissues. *Cln3* transcript abundance of *Cln3*^*Q352X*^ mice was significantly lower in all tissues at 1-month of age.

	Tissue	Male			Female		
		Ratio to WT (WT = 1)	SEM	*p* value	Ratio to WT (WT = 1)	SEM	*p* value
*Cln3* transcript abundance	Cerebral cortex	0.6629	0.0696	**0.0062	0.4861	0.03157	**** < 0.0001
	Thalamus	0.5452	0.07074	**0.0017	0.3706	0.02216	*0.0316
	Cerebellum	0.8023	0.3351	0.6064	0.8707	0.3023	0.6901
	Brainstem	0.5549	0.166	*0.0465	0.3361	0.04289	**** < 0.0001
	Eye	0.9475	0.04826	0.6637	0.6683	0.05344	*0.0322
	Heart	0.9819	0.2918	0.954	0.3409	0.05461	***0.0002
	Lung	0.4116	0.1583	*0.0127	0.4777	0.1165	**0.0052
	Liver	0.4301	0.05969	***0.0002	0.1991	0.06627	0.0886
	Kidney	0.5038	0.04997	***0.0002	0.3799	0.1062	**0.0013
	Spleen	0.3524	0.0772	**0.0093	0.5022	0.1768	0.102
	Skeletal muscle	0.7366	0.1624	0.1693	0.06647	0.0123	0.3251
PPT1 enzyme activity	Cerebral cortex	0.7661	0.1278	0.1667	0.8603	0.1283	0.4239
	Thalamus	1.094	0.03032	0.1569	0.8216	0.2833	0.6876
	Cerebellum	1.019	0.06252	0.7108	1.169	0.2876	0.7012
	Liver	0.4493	0.04273	***0.0010	0.3517	0.044	**0.0041
	Kidney	0.8651	0.08678	0.7256	1.011	0.1025	0.1079
TPP1 enzyme activity	Cerebral cortex	1.433	0.1849	0.0882	1.306	0.1494	0.1818
	Thalamus	2.302	0.1929	*0.0108	2.649	0.8586	0.1125
	Cerebellum	2.141	0.03622	**** < 0.0001	4.963	1.348	*0.0339
	Liver	0.9617	0.08109	0.6927	0.7713	0.05264	0.0693
	Kidney	1	0.05606	0.9894	1.119	0.1053	0.3733

Fluorogenic PPT1 and TPP1 enzyme activity assays performed on tissue from 1 month old *Cln3*^*Q352X*^ and WT mice present variable PPT1 levels in several tissues of *Cln3*^*Q352X*^ mice, with a significant decrease in the liver. An increase in TPP1 enzyme activity was significantly observed in the thalamus and cerebellum in *Cln3*^*Q352X*^ mice. Unpaired *t* test, mean ± SEM, N = 2–4/sex/genotype.

*p < 0.05; **p < 0.01; ***p < 0.001; ****p < 0.0001.

Loss of CLN3 has been shown to affect lysosomal function^21–23^, and several recent articles have shown that various lysosomal enzymes, including PPT1 (protein product of *CLN1*) and TPP1 (protein product of *CLN2*), are altered in *Cln3*^*Δ7/8*^ mice^24,25^. To examine whether this was the case in *Cln3*^*Q352X*^ mice, PPT1 and TPP1 enzyme activity assays were performed, showing decreased PPT1 activity in the liver of *Cln3*^*Q352X*^ mice and increased TPP1 activity in the thalamus and cerebellum (Table 2), consistent with previous reports^26,27^.

### *Cln3*^*Q352X*^ mice have typical pathological progression of Batten disease hallmarks

Wildtype and homozygous *Cln3*^*Q352X*^ mice were sacrificed at 6 and 21 months of age (early and late disease stage) to determine whether the mouse model displayed the typical pathological progression reported in CLN3 patients and other *Cln3* models. In particular, astrocyte activation (GFAP^+^), microgliosis (CD68^+^), and accumulation of lysosomal storage material (mitochondrial ATP synthase subunit C) were examined in the brain as hallmarks of CLN3 disease progression^28–32^. As regional pathogenesis starts in the somatosensory cortex and thalamus, and male and female patients have shown differences in disease progression, assays were analyzed across gender in the somatosensory barrel field (S1BF) cortex and the ventral posteromedial/ventral posterolateral (VPM/VPL) nuclei of the thalamus^4,33^.

**Figure 2:**
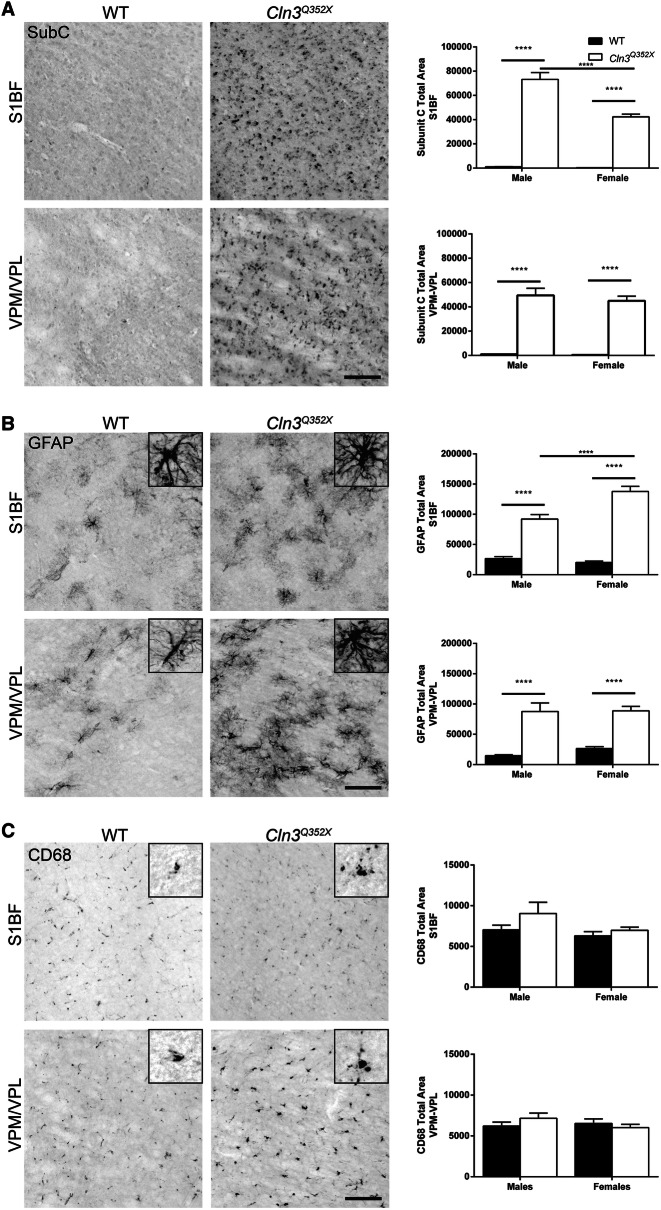
*Cln3*^*Q352X*^ mice exhibit common pathological hallmarks at 6-months of age. Immunohistochemistry on 6 month tissue showed significant increases in subunit C immunoreactivity (**A**) and GFAP immunoreactivity (**B**) in multiple brain regions for both male and female *Cln3*^*Q352X*^ mice. Microglial activation, as reflected by CD68 immunoreactivity (**C**), was decreased in the somatosensory cortex (S1BF) and thalamus (VPM/VPL) of male *Cln3*^*Q352X*^ mice as well as the S1BF of female *Cln3*^*Q352X*^ mice. A slight increase in microglial activation was observed in the VPM/VPL of female *Cln3*^*Q352X*^ mice**.** Two-way ANOVA, Fisher’s LSD post-hoc. Mean ± SEM, ****p < 0.0001. All images are representative of male mice. Scale bar = 100 µm.

**Figure 3:**
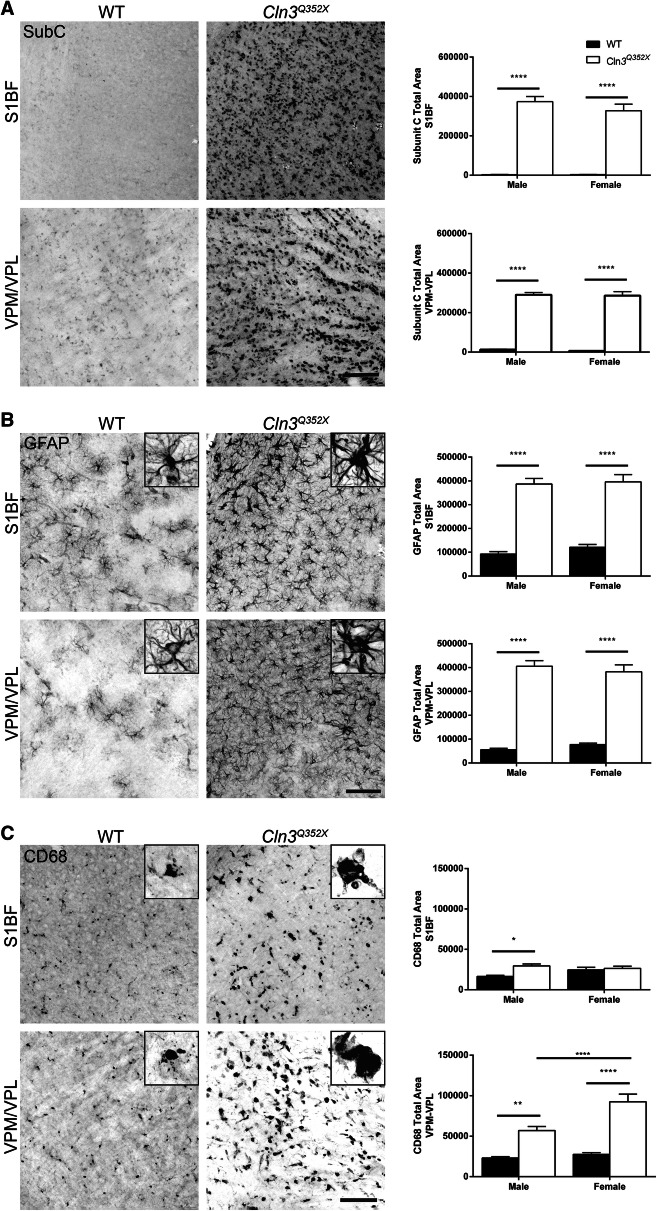
*Cln3*^*Q352X*^ mice display increased pathological hallmarks at 21-months of age. Immunohistochemistry on 21 month tissue showed significantly higher subunit C immunoreactivity (**A**) and GFAP immunoreactivity (**B**) in multiple brain regions for both male and female *Cln3*^*Q352X*^ mice. Microglial activation, revealed by CD68 immunoreactivity (**C**), is increased all brain regions for *Cln3*^*Q352X*^ mice of both sexes, except for the S1BF of female *Cln3*^*Q352X*^ mice. Two-way ANOVA, Fisher’s LSD post-hoc. Mean ± SEM, *p < 0.05; **p < 0.01; ****p < 0.0001. WT images are representative of female mice. Mutant images are representative for male mice (SubC, CD68) and female mice (GFAP). Scale bar = 100 µm.

At 6 months, subunit C accumulation was already significantly present in both the S1BF and the VPM/VPL of *Cln3*^*Q352X*^ mice while wildtype controls showed extremely minor amounts of positive immunoreactivity (Fig. 2A). Astrocytosis was also prominent at 6 months with females showing increased values in the S1BF (Fig. 2B). Additionally, there were qualitative differences in astrocyte morphology between wildtype and *Cln3*^*Q352X*^ mice, with *Cln3*^*Q352X*^ astrocytes appearing slightly more hypertrophic and ramified. There was no significant difference, however, in microglial activation at this early 6 month timepoint (Fig. 2C). Pathology was widespread and significantly exemplified at 21 months, which is near end stage of a normal mouse lifespan. Subunit C accumulation was evident throughout the brain, increasing in both S1BF and VPM/VPL regions with no apparent sex differences (Fig. 3A). Astrocytosis followed a similar trend in both regions with obvious differences in morphology as *Cln3*^*Q352X*^ displayed extreme hypertrophy and ramification when compared to wildtype animals(Fig. 3B). Additionally, microglial activation was significantly elevated in *Cln3*^*Q352X*^ mice at 21 months in the VPM/VPL for both sexes while only males were significantly altered in the S1BF compared to their wild type counterparts (Fig. 3C).

### Behavioral evaluation of *Cln3*^*Q352X*^ mice reveals sex-dependent motor coordination deficits

**Figure 4:**
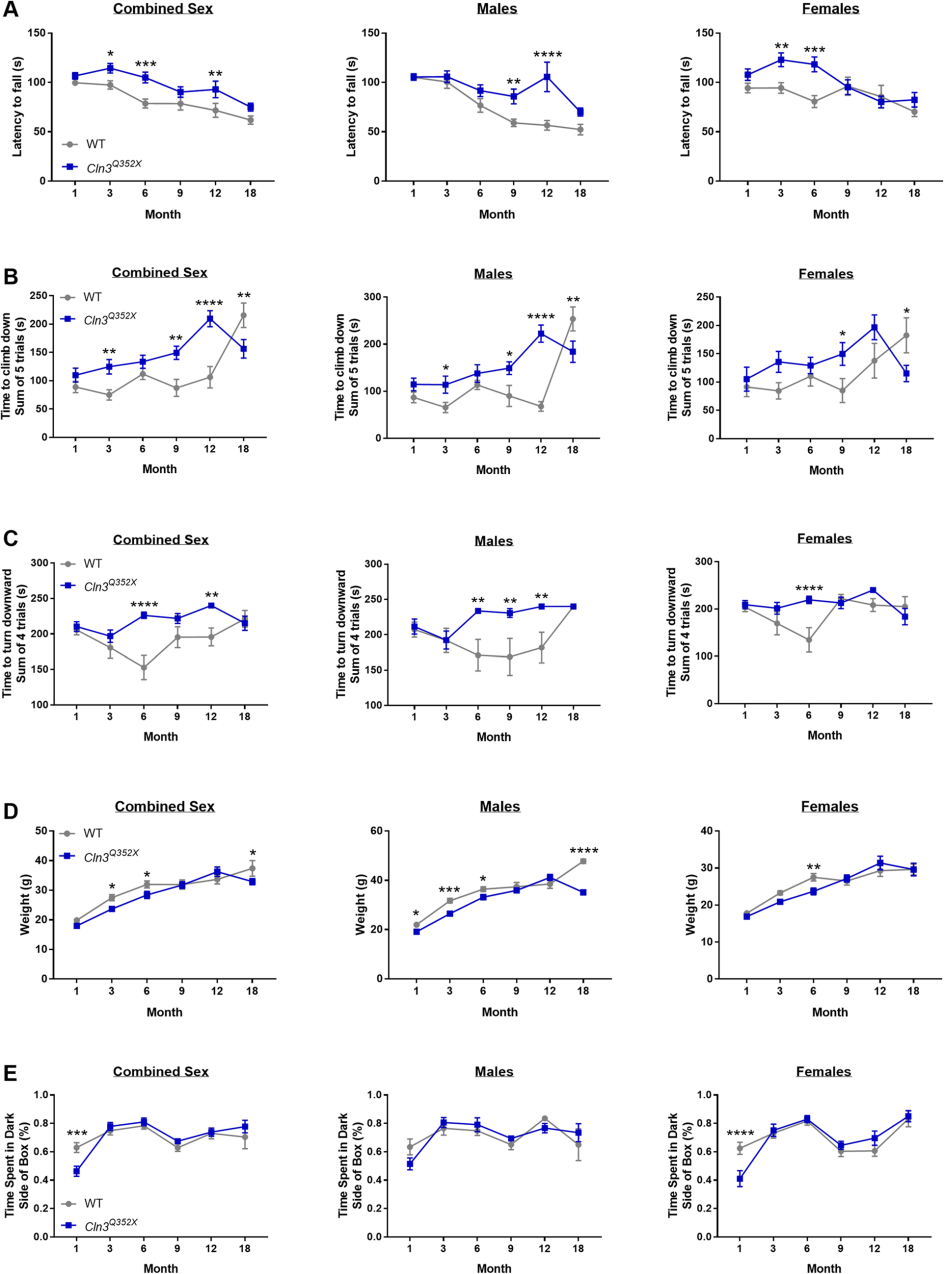
*Cln3*^*Q352X*^ mice display altered motor skills. (**A**) Male *Cln3*^*Q352X*^ mice exhibited an increased latency to fall at all time points on the rotarod, with significance at 9 and 12 months of age. Female *Cln3*^*Q352X*^ mice also displayed an increased latency to fall at all time points except 12 months, with significance at 3 and 6 months of age. (**B**) On the climb down test, an increased latency was observed for male *Cln3*^*Q352X*^ mice at 3, 9, and 12 months of age and a decreased latency at 18 months of age compared to male WT mice . Female *Cln3*^*Q352X*^ mice showed an increased latency at 9 months of age and a decreased latency at 18 months compared to female WT mice. (**C**) Time to turn downward test showed an increased latency in male *Cln3*^*Q352X*^ mice at 6 and 12 months of age compared to male WT mice. Female *Cln3*^*Q352X*^ mice only showed an increased latency at 6 months of age compared to female WT mice. (**D**) Significant differences in weight were observed at multiple time points. (**E**) WT and *Cln3*^*Q352X*^ mice spend the majority of their time in the dark side of the light/dark box with only one significant difference in female mice at 1 month of age. Two-way ANOVA, Fisher’s LSD post-hoc. Mean ± SEM, *p < 0.05; **p < 0.01; ***p < 0.001; ****p < 0.0001.

While other *Cln3* mouse models are notorious for their limited and inconsistent behavior deficits, we sought to thoroughly characterize any motor coordination deficits that may be present in *Cln3*^*Q352X*^ mice. When assessed on an accelerated rotarod test, *Cln3*^*Q352X*^ mice generally performed similarly to their wild type counterparts, and in some instances performed better compared to wild type mice (Fig. 4A). When assessed for motor coordination deficits using another assay, a modified vertical descent test, male *Cln3*^*Q352X*^ mice showed significant difficulties in climbing down and turning down the pole (Fig. 4B,C). These deficits were prominent from 3 months of age, with wild type males performing poorly at the test as they aged. Female *Cln3*^*Q352X*^ mice, however, showed inconsistent deficits in these assays.

Lastly, in order to control for weight and anxiety as confounding factors in behavior results, mice were weighed at each time point and subjected to a light–dark test to assess their preference for dark areas. *Cln3*^*Q352X*^ male mice weighed less than their wildtype cohorts at 3, 6 and 18 months, while *Cln3*^*Q352X*^ female mice weighed less only at 6 months (Fig. 4D). Any weight disadvantages experienced by wildtype mice would be most prominent at these time points, which is not seen in the rotarod test, as there were no differences between wildtype and *Cln3*^*Q352X*^ mice at these time points. However, heavier wildtype mice may have been disadvantaged in the 18 month vertical descent test, as male wildtype mice took significantly longer to descend the pole when compared to the lighter *Cln3*^*Q352X*^ male mice. When assessed for anxiety and preference for dark areas, both groups spent the majority of their time in the dark side of the box with only one significant difference in female *Cln3*^*Q352X*^ mice at 1 month of age (Fig. 4E), indicating that anxiety likely did not contribute to the results of the other behavioral tests.

## Discussion

Batten disease is a lysosomal storage disorder that, while individual subtypes are rare, represents the most common pediatric neurodegenerative disease worldwide. CLN3 Batten disease results from homozygous mutations in CLN3, which are present in ~ 80% of individuals inflicted with Batten disease. Although the function of the CLN3 protein is unknown, the pathogenic progression of the disease is well established in both human patients and animal models. Common symptoms of CLN3 Batten disease include progressive vision loss, neurocognitive decline, behavioral changes, and motor deficit with symptom onset beginning around ~ 4–8 years and death occurring at ~ 20 years. In mouse models of CLN3 Batten disease, disease progression results in whole brain atrophy, including region-specific atrophy in the cortex, hippocampus, thalamus, and cerebellum. Additionally, mice present with pathological hallmarks including reactive gliosis, neuroinflammation, and accumulation of ASM. However, while *Cln3* mouse models recapitulate some motor and pathological signs of the disease, the visual and cognitive aspects of the disease in human patients do not develop.

While the genetic knockout mouse models have been beneficial for studying the selective pathogenesis of Batten disease, recently, tailored mouse models have been generated to develop therapies to individual mutations. Generation of novel mouse models from patient mutations of Batten disease present with many of the same neuronal and visual phenotypes seen in patients^29,31,34–59^. Regional-specific vulnerability of cortical, hippocampal, thalamic, and cerebellar regions of the CNS have been identified in many of the murine models of Batten disease^29,31,37,38,44,60,61^. These models also help in longitudinal identification of pathological hallmarks and detection of glial activation before neuronal changes occur.

*Cln3*^*Q352X*^ displayed the typical pathological progression of disease hallmarks as other Cln3 mouse models. ASM was evident in the somatosensory cortex and the thalamus as early as 6 months (Fig. 2A) and became widespread and extreme at 21 months of age (Fig. 3A). Similarly, reactive astrocytosis was prevalent at 6 months (Fig. 2B) which significantly worsened at 21 months with clear morphological differences between wild type and *Cln3*^*Q352X*^ mice (Fig. 3B). While microglial activation (CD68) at 6 months was not significant in either of the brain regions analyzed, it was significantly increased at 21 months in *Cln3*^*Q352X*^ mice, and it *Cln3*^*Q352X*^ mice appeared to have hypertrophic microglia at this stage in disease (Fig. 3C).

We have previously demonstrated that the neurological deficits in the *Cln3*^*-/-*^ and *Cln3*^*Δex7/8*^ mouse models of juvenile CLN3 disease strongly depend on the genetic background and gender ^34^, and female CLN3 patients have reported more severe symptoms^33^. Therefore, the age-dependent disparate behavioral test results of male and female *Cln3*^*Q352X*^ mice are not surprising, though notably female *Cln3*^*Q352X*^ mice lacked a consistent, more severe phenotype. In the vertical pole test, *Cln3*^*Q352X*^ mice showed age dependent motor deficits compared to wild type mice. In the accelerating rotarod test, however, *Cln3*^*Q352X*^ mice performed better than wild type mice at certain ages. These surprising results are not unique to *Cln3*^*Q352X*^ mice, as we have previously shown that while *Cln3*^*Δex7/8*^ female mice displayed motor deficits in the vertical pole test, they performed markedly better than their wild type and *Cln3*^*−/−*^ counterparts in the rotarod test^34^.

Although, decreased *Cln3*^*Q352X*^ transcript levels were seen in several organs and brain regions of both male and female *Cln3*^*Q352X*^ mice (Table 2), no statistically significant reduction of *Cln3*^*Q352X*^ mRNA was detected in the cerebellum, muscle, heart and eye of males, and the cerebellum, muscle, spleen and liver of females. It has been shown that inter-tissue and sex variations exist in the efficiency of nonsense-mediated mRNA decay in mice, and therefore, it is possible that these variations account for the differences seen in our model^62,63^. It has previously been shown that the activity of the soluble lysosomal enzyme, TPP1 (product of the *CLN2* gene) is markedly increased in the brain of patients with juvenile CLN3 disease and also in the brain of *Cln3*^*-/-*^ mice^24–26^. In *Cln3*^*Q352X*^ mice, however, TPP1 activity, was only significantly elevated in the thalamus and cerebellum, perhaps suggesting that the *Cln3*^*Q352X*^ mutation does not affect lysosomal function as severely as the most common disease-causing deletion (*Cln3*^*Δex7/8*^) or the complete lack of *Cln3* (*Cln3*^*-/-*^). Importantly, the clinical presentation of the *Cln3*^*Q352X*^ patient mutation has not been reported, and it is therefore unclear whether the point mutation brings about less severe cellular manifestations and, as a consequence, disease course.

Taken together, we show pathological and behavioral characterization of a novel point mutant *Cln3*^*Q352X*^ mouse model, which shows progression of pathological symptoms similar to the common *Cln3*^*Δex7/8*^ mouse model. Beneficially, *Cln3*^*Q352X*^ model enables the screening of read-through and nonsense suppression therapies in the future, that if tested in the common *Cln3*^*Δex7/8*^ model would be confounded by the presence of novel amino acids. Therefore, this novel model shows its utility as a pathological model of CLN3 Batten disease, and its potential as a tool for screening tailored genetic therapies in the future.

## Methods

### Ethics statement

All animal research performed for this manuscript followed National Institute of Health (NIH) and Sanford Research Institutional Animal Care and Use Committee (IACUC) guidelines. The animal protocol for this study was reviewed and approved by the Sanford Research IACUC.

### Mouse generation

*Cln3*^*Q352X*^ mice were generated by Applied StemCell Inc. (CA). Using CRISPR technology, a nonsense mutation was introduced in exon 16 (CAG > TAG) causing glutamine352 (Q) to be replaced with a premature stop codon. Addition of a silent mutation (CTC > TTG) directly before the nonsense mutation was necessary to generate the mouse line and codes for the same amino acid (leucine). Specific guide RNAs, oligo donors, and Cas-9 vectors are described in Fig. 1 and Table 1. gRNA non-homologous end joining efficiency was determined by Applied StemCell Inc. using a SURVEYOR assay on PCR products (Transgenomic Inc., #706020), and cloned and F1 generations were confirmed by sequencing chromatograms. F2 generations were sequence confirmed using a quantitative real-time polymerase chain reaction assay (Forward primer: 5′- ACGGTTCACCTGGGTCCTA-3′; Reverse primer: 5′-GAGGTGGGTGTGAACAAACAG-3′; Reporter 1(VIC): 5′-CACTCAGTACCTGGAGCAG-3′; Reporter 2(FAM): 5′- CACTCAGTACCTACAACAG-3′) from Thermo Fisher Scientific. Thermal profile for qRT-PCR assay included: 1) 95 °C for 15 min. (1 cycle) and 2) 95 °C for 15 s. and 60 °C for 1 min. (40 cycles). *Cln3*^*Q352X*^ mice were generated and maintained on the C57BL/6J (Stock #000664) background and were characterized in comparison to wild type C57BL/6J mice maintained in our mouse colony. All mice were housed, cared for, and used under the procedures developed by the NIH and the direction of the Sanford Research IACUC (Protocol #: 129-03-20B). Mice were housed in separately vented microisolator cages (maximum of five mice per cage) with access to food and water without restrictions, with the room maintained at a 14 h dark: 10 h light cycle. Mice were fed with the Teklad Global 2918 diet (Harlan Laboratories, IN) and their drinking water was tap water.

### Behavioral characterization

All behavior tests were performed during the 10 h light cycle. Mice performed three different tests to determine the impact of the *Cln3*^*Q352X*^ mutation on parameters of performance such as motor coordination, spatial awareness, and anxiety-like behavior. Mice were divided into two behavioral cohorts to minimize motor learning and repeated measures testing. Behavioral tests were performed on the same groups of male and female *Cln3*^*Q352X*^ mice and wild type mice in Cohort 1: 1, 3, and 6 months of age (n = 12 /sex/genotype) and Cohort 2: 9, 12, and 18 months of age (n = 11 of each sex/genotype). Before behavioral tests were performed, mice were allowed a 20 min acclimation period to their testing environment.

### Rotarod

Motor coordination was assessed using an accelerating rotarod test as previously described^64^. In brief, a Rota Rod Rotamex-5 (Columbus Instruments, OH) was utilized. Parameters for the rotarod test were as follows: start speed: 0 rotations per minute (rpm); end speed: 48 rpm, acceleration: 0.2 rpm per second, and total allotted test time: 240 s. Mice underwent three consecutive training runs on the rotarod before a 1.5 h rest period. After the rest period, mice were tested on the rotarod for three test trials comprising three consecutive testing runs with 15 min of rest between each trial. Latency to fall was analyzed as an average of the nine test runs for each mouse.

### Modified vertical pole test

In order to test motor coordination and spatial awareness, a modified vertical pole test was performed as previously described^64^. Briefly, the vertical pole utilized a threaded metal pole attached to a padded base and consisted of two tests: (1) climb down: mice were placed at the top of the and timed until they climbed down to the base of the; and (2) turn downward: mice were placed at the top of the pole facing up and timed until they turn downward. For the climb down test, mice were given 60 s to climb down the pole, performing the test five consecutive times. During the turn downward test, mice were again given 60 s to turn around and face downward, performing the test four consecutive times. Mice that fall off the pole in either test were given a score of 60 s. The total sum of time in the trials for each test and for each mouse was calculated for analysis.

### Light/dark box

Mice were placed individually into a box with a light side and a dark side (Stoelting Co., IL) and allowed to roam freely for 10 min. Mouse movement was recorded and tracked using ANYmaze equipment and software (Stoelting Co., IL). Software data was used to determine the amount of time spent in each area of the box. The time spent in the dark box is proportional to anxiety, as anxious mice spend little time in the light box.

### Tissue collection and preparation

For enzyme analysis and transcript abundance, *Cln3*^*Q352X*^ mice and wild type mice (n = 4/sex/genotype) were euthanized using a carbon dioxide chamber at 1 month of age. Following euthanasia, mice were flushed with 10 ml of sterile phosphate buffered saline solution by cardiac perfusion. Collected tissues were flash frozen on dry ice, and included the cerebral cortex, cerebellum, striatum/thalamus, liver, and kidney. All flash frozen tissues were stored at − 80 °C until analysis. Tissues for enzyme analysis were homogenized, processed, and extracted using a Maxwell 16 LEV simplyRNA tissue kit (Promega), while tissues for enzyme analysis were homogenized in protein lysis buffer. Homogenization of tissues was performed in a Bertin Technologies Precellys 24 homogenizer using 2-ml tubes containing zirconium beads. For histological analysis*, Cln3*^*Q352X*^ mice and wild type mice (n = 6/sex/genotype) were euthanized at 6 and 21 months of age. Following euthanasia, animals were flushed with 10 ml of sterile phosphate buffered saline solution followed by a 10-ml perfusion of 4% paraformaldehyde in PBS both via cardiac perfusion. Extracted brains were placed into scintillation vials containing 4% paraformaldehyde in PBS. Brains were stored at 4 °C with PBS containing 0.02% sodium azide replacing the 4% paraformaldehyde solution after 24 h fixation. Brains were set in 5% low melting agarose and sliced coronally into 50 μm sections using a Leica VT1000S vibratome in a 1:6 serial collection.

### Protein sample preparation

Protein sample preparation was performed as previously described^27^. Briefly, tissue were cut and placed into 2-ml tubes containing zirconium beads and 500 μl of protein lysis buffer and homogenized using a Bertin Technologies Precellys 24 homogenizer. Homogenized samples were centrifuged at 12,000*g* for 10 min with supernatant sample transfered to 1.5-ml microcentrifuge tubes and storage at − 80 °C. Total protein concentrations were determined using the Pierce 660 nm Protein Assay (Thermo Fisher Scientific).

### Batten-related lysosomal enzyme activity assays

Evaluation of TPP1 (tripeptidyl peptidase 1) and PPT1 (palmitoyl protein transferase 1) enzyme activities were completed using samples from *Cln3*^*Q352X*^ and wild type mice as previously described^14,27^. Assays were performed on 10 μg of protein sample per well. For the TPP1 enzyme assay, protein sample was accompanied by 40 μl of 250 μM Ala-Ala-Phe-7-amido-4-methylcoumarin diluted in substrate buffer (150 mM NaCl; 0.1% Triton X-100; 100 mM sodium acetate; pH 4.0). PPT1 enzyme assay included protein sample accompanied by 20 μl of PPT1 substrate (0.64 mM 4-methylumbelliferyl-6-thiopalmitoyl-β-glucoside; 15 mM dithiothreitol with 2.3 mg/ml bovine serum albumin and 0.09% sodium azide; 0.2 M disodium phosphate with 0.1 M citric acid buffer; 0.945 mg/ml β-glucosidase; pH 4.0). Enzyme activity assay plates were incubated in the dark for 1 h at 37 °C, then 200 μl of stop buffer was added (TPP1 Stop Buffer: 0.1 M monochloroacetic acid, 0.13 M NaOH, 0.1 M acetic acid, pH 4.3; PPT1 Stop Buffer: 0.5 M sodium bicarbonate with 0.5 M sodium carbonate, 0.025% Triton X-100, pH 10.7). Fluorescence was measured using a BioTek Cytation 3 (TPP1: Excite 380 nm/Emit 460 nm; PPT1: Excite 355 nm/Emit 460 nm). With background fluorescence being accounted for, relative fluorescence was measured as a percentage of wild type fluorescence.

### RNA sample preparation and reverse transcription for cDNA synthesis

RNA and cDNA sample preparation were performed as previously described^27^**. **RNA sample concentrations were calculated and cDNA synthesis was executed on 1 μg of total RNA using GoScript Reverse Transcription System (Promega). RT-PCR Reactions were performed on an Applied Biosystems Veriti Thermal Cycler according to manufacturer’s protocol. Samples were stored at − 20 °C.

### Quantitative polymerase chain reaction

Using nuclease-free water, cDNA samples were diluted 1:20. Quantitative polymerase chain reactions were performed using Absolute Blue Q-PCR Mix (Thermo Fisher Scientific). *Cln3* (Forward primer: 5′-TGGAGACCAGTGACAAGCA-3′; Reverse primer: 5′-TCAAGGGAGGTGACAGAGGA-3′) and *Gapdh* expression (Forward primer: 5′-ACCACAGTCCATGCCATCAC-3′; Reverse primer: 5′-TCCACCACCCTGTTGCTGTA-3′) were quantified. Reactions were performed in a Stratagene Mx3005P (Agilent Technologies) under the following conditions: 1) 95 °C for 15 min. (1 cycle) and 2) 95 °C for 15 s.; 60 °C for 1 min. (40 cycles). 2^-*ΔΔCT*^ method was used to analyze relative fold expression^65^.

### Immunohistochemistry

Three brain sections per animal were selected to contain the ventral posteromedial (VPM)/ventral posterolateral (VPL) nuclei of the thalamus and the somatosensory barrel field (S1BF) cortex. Immunohistochemistry was performed as previously described^66^. Briefly, free floating brain sections were incubated in 1% hydrogen peroxide in Tris-buffered saline (TBS) for 20 min to block endogenous peroxidase activity followed by 3 washes in TBS. Sections were then blocked in 15% goat serum-containing TBS-T (TBS with 0.3% Triton X-100) for 30 min followed by primary antibody solution overnight at 4 °C. Primary antibodies included: anti-CD68 (BioRad AbD Serotec, MCA1957; 1:2,000), anti-GFAP (Dako, Z0334; 1:8,000), and anti-ATP synthase C (Abcam, ab181243, 1:1,000) diluted in TBS-T+ 10% goat serum. Secondary antibodies included: biotinylated anti-rat and anti-rabbit IgGs (Vector Labs, BA-9400, BA-1000; 1:2000) diluted in TBS-T+ 10% goat serum. An ABC amplification kit (Vector Labs) was used for 2 h followed by 0.05% DAB solution until visible reaction had occurred.

### Image acquisition and analysis

All microscope slides were scanned on a Leica DM6000B slide scanning microscope at 20X. Images were extracted in 2,400 × 2,400 pixel fields from appropriate regions and analyzed using an image threshold analysis using ImageJ (version 1.51). High-resolution images for figures were taken on a Nikon NiE microscope.

### Statistical analysis

Statistical analyses were performed using GraphPad Prism (v6.04). Male and female *Cln3*^*Q352X*^ mice were compared to their same-sex cohort for all statistical analyses using two-way ANOVA with Fisher’s LSD and outlier removal using the ROUT method, Q = 1%.
